# Resource shifts in Malagasy dung beetles: contrasting processes revealed by dissimilar spatial genetic patterns

**DOI:** 10.1111/j.1461-0248.2008.01239.x

**Published:** 2008-11

**Authors:** Ilkka Hanski, Helena Wirta, Toshka Nyman, Pierre Rahagalala

**Affiliations:** 1 Department of Biological and Environmental Sciences, University of HelsinkiViikinkaari 1, PO Box 65, FI-00014, Helsinki, Finland; 2 Département d’Entomologie, Faculté des Sciences, Université d’AntananarivoAntananarivo, B.P. 906, Madagascar

**Keywords:** Cattle dung, dung beetle, genetic diversity, host shift, Madagascar, range expansion, resource competition, resource shift

## Abstract

The endemic dung beetle subtribe Helictopleurina has 65 species mostly in wet forests in eastern Madagascar. There are no extant native ungulates in Madagascar, but three *Helictopleurus* species have shifted to the introduced cattle dung in open habitats in the past 1500 years. *Helictopleurus neoamplicollis* and *Helictopleurus marsyas* exhibit very limited cytochrome oxidase subunit 1 haplotype diversity and a single haplotype is present across Madagascar, suggesting that these species shifted to cattle dung in a small region followed by rapid range expansion. In contrast, patterns of molecular diversity in *Helictopleurus quadripunctatus* indicate a gradual diet shift across most of southern Madagascar, consistent with somewhat broader diet in this species. The three cattle dung-using *Helictopleurus* species have significantly greater geographical ranges than the forest-dwelling species, apparently because the shift to the currently very abundant new resource relaxed interspecific competition that hinders range expansion in the forest species.

*Ecology Letters* (2008) 11: 1208–1215

## Introduction

Shifts in resource use often punctuate both the ecological and the evolutionary dynamics of species. Resource shifts facilitate sympatric (Bush 1969; Harrison 1991; Schwarz *et al.* 2005) and allopatric speciation (Huyse *et al*. 2005), and are typically involved in adaptive radiations of entire species assemblages (Schluter 2000). Resource shifts may lead to range expansion and changes in population dynamics, as exemplified by the European corn borer moth (*Ostrinia nubilalis*), which has two sympatric host races in Europe, one feeding on *Artemisia vulgaris* and the other using maize. The latter race must have evolved through host shift following the introduction of maize to Europe 500 years ago (Calcagno *et al*. 2007). The host shift has allowed the maize-using race to greatly expand its range, even across the Atlantic to North America, where the European corn borer has become a serious pest. Host shift-induced range expansions may be common, but examples of it tend to be rather anecdotal.

Madagascar has a unique biota with an exceptionally high level of endemism due to its ancient isolation (Myers *et al.* 2000; Briggs 2003; de Wit 2003). Malagasy communities lack entire families and even orders of animals that are common on the African mainland, but which never managed to colonize Madagascar. In mammals, there are no extant native ungulates, and the largest Malagasy herbivores are primates (lemurs). This makes a big difference to dung beetles, which we have studied. Most Malagasy dung beetles belong to the subtribe Helictopleurina and to the tribe Canthonini, of which the former is endemic and started to radiate with lemurs 37–23 Ma ago (Wirta *et al.* 2008). On the African continent, the majority of dung beetles occur in grasslands using the dung of large-bodied herbivores and omnivores (Hanski & Cambefort 1991), but in Madagascar practically all native species occur in forests, where they use mostly lemur excrements and carrion (Koivulehto 2004; Viljanen 2004; Wirta *et al.* 2008).

The range of resources available to Malagasy dung beetles changed greatly following the human colonization 2300 years ago (Burney *et al.* 2004). Humans caused the extinction of the Malagasy megafauna, including all large-bodied (> 10 kg) species of lemurs, pigmy hippopotami, giant tortoises and elephant birds (Burney *et al.* 2004). Dung beetles are not expected to have made much use of the dung of hippopotami, tortoises and elephant birds, based on results for the use of the dung of related species in mainland Africa (Gittings & Giller 1998; Davis *et al.* 2002), but the extinction of large-bodied lemurs was significant, although to some extent humans themselves replaced the primates that went extinct. Potentially, even more significant to dung beetles was the introduction of cattle about 1500 years ago (Burney *et al.* 2004). There are currently 7 million bovines across the island, mostly in open habitats but low-density feral cattle occurs also in many forested regions (P. Rahagalala, H. Viljanen and I. Hanski, unpublished data).

Cattle and comparable ungulate dung teem with dung beetles both in forests and savannas in the tropics, with local communities often exceeding 50 species (Hanski & Cambefort 1991). In Madagascar, the situation is very different. In wet forests, not a single dung beetle species uses cattle dung as the primary food resource, and altogether there are only a few species that use cattle dung at all (Rahagalala *et al.*, unpublished data). In open habitats, there are *c*. 20 cattle dung-using species, including introduced species and several small Aphodiidae (Rahagalala *et al.*, unpublished data). The local communities have 5–10 species, which is almost an order of magnitude less than in comparable communities in mainland Africa (Rahagalala *et al.*, unpublished data).

The well-studied endemic subtribe Helictopleurina has 65 species, of which 71% occur in wet forests in eastern Madagascar, 14% in dry forests in western Madagascar and 7% in various forests across the country (Wirta *et al.* 2008). This leaves five species, which are exceptional and occur in cattle dung in open habitats: *Helictopleurus quadripunctatus*, *Helictopleurus marsyas*, *Helictopleurus neoamplicollis*, *Helictopleurus littoralis* and *Helictopleurus sinuatocornis.* These species must have shifted to cattle dung in the past 1500 years since the introduction of cattle to Madagascar. At present, *H. quadripunctatus*, *H. marsyas* and *H. neoamplicollis* are very common, *H. littoralis* is less common, while *H. sinuatocornis* is an apparently uncommon high-altitude species (Wirta *et al.* 2008). For lack of material, we do not consider *H. sinuatocornis* further in this paper (but see Wirta *et al.* 2008).

The three common *Helictopleurus* species have maximally large geographical ranges, as they occur across Madagascar, and their ranges are in fact significantly greater than the ranges of the forest-dwelling *Helictopleurus* species (Wirta *et al.* 2008). The biology of the cattle dung-using *Helictopleurus* does not indicate whether the shift to cattle dung occurred in one (small) region, followed by range expansion, or whether species that had originally exceptionally large ranges have managed to shift to cattle dung. Here, we attempt to answer this question by examining haplotype diversities and spatial distributions in the variable cytochrome oxidase subunit 1 (CO1) locus in the cattle dung-using species and in related forest-dwelling species. Rapid range expansion leaves the signature of reduced molecular diversity. For instance, many northern temperate taxa that now occur in glaciated regions show reduced molecular diversity in the north compared with the areas in the south that were not glaciated (Hewitt 1996, 1999; Taberlet *et al.* 1998). Painter *et al.* (2007) described an extreme example, the old-growth forest specialist beetle *Pytho kolwensis*, which colonized Europe from northern Asia following the last glacial period. A single CO1 haplotype dominates in Europe, whereas molecular diversity is high in northern China, in the putative glacial refugium. Similarly, speciation via host shift is expected to result in significantly lower level of genetic variation in populations using the derived host because of founder effect (Harrison 1991). Host shift-induced range expansion should additionally produce no spatial genetic structure in the species that shifted to a new host or new resource, which is the genetic signature we are looking for. The control group is comprised by the forest-dwelling species, which are expected to have retained their current geographical ranges for a long time or have reduced ranges due to deforestation (Hanski *et al.* 2007).

## Material and Methods

### Taxa

We obtained samples of seven *Helictopleurus* species across their ranges in Madagascar, including three sets of closely related species (for molecular phylogeny see Fig. 4 in Wirta *et al*. 2008): *H. quadripunctatus* (54 individuals from 19 localities), *Helictopleurus unifasciatus* (29, 7) and *Helictopleurus perrieri* (11, 3); the sister species pair *H. marsyas* (44, 17) and *Helictopleurus nicollei* (5, 1); and the sister species pair *H. neoamplicollis* (100, 31) and *H. littoralis* (12, 7). Of the seven species, *H. unifasciatus, H. perrieri* and *H. nicollei* are forest-inhabiting species, while the rest occur in cattle dung in open habitats. Additionally, we have included for comparison one forest-dwelling Canthonini, *Nanos clypeatus*, for which comparable data (5–10 individuals from eight localities) are available (Wirta 2008). These latter data include material for the nominal species *Nanos dubitatus*, which appears to be conspecific with *N. clypeatus* (Wirta 2008).

### DNA extraction and sequencing

Beetles were preserved in 95% ethanol or dried prior to DNA extraction. One to five individuals from each locality, depending on the availability, were sequenced for the mitochondrial CO1. Sequences have been submitted to GenBank (accession numbers EU429040 EU429294). The protocols used for DNA extraction, amplification and sequencing are the same as in Orsini *et al.* (2007), with the addition of 1 μm of trehalose to improve amplification (Spiess *et al.* 2004) in the reaction volume of 20 μL. We used the primers Pat (5′-TCCAATGCACTAATCTGCCATATTA-3′) and Jerry (5′-CAACATTTATTTTGATTTTTTGG-3′) (Simon *et al.* 1994).

### Genetic and phylogeographic analyses

The sequences were aligned with clustalw (Thompson *et al.* 1994) and adjusted by eye. Molecular variation was examined with mega4 (Tamura *et al.* 2007). Haplotype networks were constructed with the program TCS (Clement *et al.* 2000) using the connection limit of 95%.

## Results

The sister species pair *H. neoamplicollis* and *H. littoralis* shared the same haplotypes, including populations in the two localities in which they occurred together in our samples. This result implies ongoing gene flow throughout the ranges of the species. *Helictopleurus littoralis* is distinguished by smaller body size and some subtle morphological differences (Montreuil 2005). However, as there are no known ecological differences between the species, and as the slight differences in morphology may be related to body size difference, it is likely that *H. littoralis* represents small individuals of a single taxon, which is here called *H. neoamplicollis.*

The six *Helictopleurus* species fall into two classes in terms of the level of molecular diversity, with 5–6% of the nucleotide sites polymorphic in *H. quadripunctatus*, *H. unifasciatus* and *H. perrieri*, while < 1% polymorphic in the remaining species (Table 1). *Nanos clypeatus* (Canthonini) shows even greater molecular diversity than the forest-dwelling *H. unifasciatus* and *H. perrieri* (Table 1).

**Table 1:** Molecular variation in CO1 in six species of *Helictopleurus* and in *Nanos clypeatus* (Canthonini)

Species	*n*	bp	V%	Pi%	S%	Diet	Habitat
*Helictopleurus marsyas*	44	688	0.44	0.15	0.29	Cattle dung	Open area
*Helictopleurus neoamplicollis*	112	688	1.02	0.15	0.87	Cattle dung	Open area
*Helictopleurus quadripunctatus*	54	688	5.81	3.34	2.47	Cattle and human dung, carrion	Open area and dry forest
*Helictopleurus nicollei*	5	688	0.00	0.00	0.00	Carrion and other dung	Wet forest
*Helictopleurus perrieri*	11	688	5.09	3.63	1.45	Carrion and other dung	Dry forest
*Helictopleurus unifasciatus*	29	688	5.96	3.63	2.33	Carrion and other dung	Dry forest
*Nanos clypeatus*	54	771	10.89	9.21	1.69	Carrion and other dung	Wet forest

*Helictopleurus littoralis* has been combined here with *Helictopleurus neoamplicollis.*

V%, Pi% and S% are the percentages of variable, parsimony informative and singleton sites, respectively.

**Figure 1:**
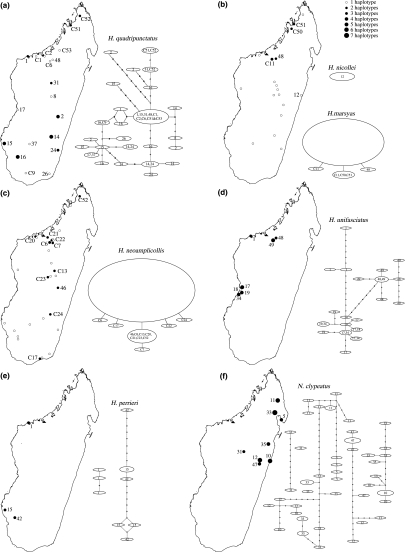
Distribution of haplotypes (maps) and haplotype networks in three cattle dung-using *Helictopleurus* species, *Helictopleurus quadripunctatus* (a), *Helictopleurus marsyas* (b) and *Helictopleurus neoamplicollis* (c), in two forest-dwelling *Helictopleurus* species, *Helictopleurus unifasciatus* (d) and *Helictopleurus perrieri* (e), and in one forest-dwelling Canthonini, *Nanos clypeatus* (f; from Wirta 2008). The size of the symbol indicates the number of different haplotypes recorded per sampling locality (see the legend in the figure).

**Figure 2:**
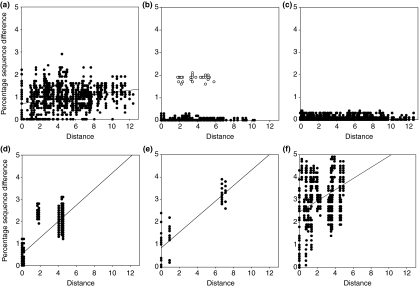
Percentage sequence difference between pairs of individuals against the corresponding geographical distance (in degrees, corresponding to 112 km at the equator). Upper row: *Helictopleurus quadripunctatus* (a), *Helictopleurus marsyas* (b) and *Helictopleurus neoamplicollis* (c). The open symbols in (b) are for pairs of individuals involving *H. marsyas* and *Helictopleurus nicollei* (see text). Lower row: *Helictopleurus unifasciatus* (d), *Helictopleurus perrieri* (e) and *Nanos clypeatus* (f; from Wirta 2008).

All three forest-dwelling species, *H. unifasciatus*, *H. perrieri* and *N. clypeatus*, show a distinct spatial structure in the occurrence of CO1 haplotypes and no single haplotype is numerically dominant (Fig. 1). There is a highly significant isolation by distance relationship in these species, with the average sequence difference between two individuals reaching *c*. 3% at the distance of 500 km (Fig. 2).

The results are strikingly different for the cattle dung-using *H. neoamplicollis* and *H. marsyas*, in which most individuals represent just a single haplotype (Fig. 1b,c) with no indication of isolation by distance (Fig. 2b,c). In both species, what little haplotype diversity there exists occurs in the same region in north-western Madagascar (Fig. 1b,c). *Helictopleurus nicollei*, the sister species of *H. marsyas*, has been sampled from a single locality on the east coast, which is essentially its current known range based on our own sampling and museum collections. Our sample includes only one haplotype for this species (Fig. 1b). There is a clear difference between *H. nicollei* and *H. marsyas*, with 13 fixed nucleotide differences (1.9%; Fig. 2b).

The third cattle dung-using species, *H. quadripunctatus*, shows patterns that are intermediate between the other cattle dung-using species and the forest species (Figs 1 and 2). *Helictopleurus quadripunctatus* has high haplotype diversity especially in southern Madagascar, but there is also one common haplotype, which is the only one in many samples within a large area in north-western Madagascar (Fig. 1a). The isolation by distance relationship is statistically significant (*P* < 0.001, *R*^*2*^ = 0.08), but the slope is only 15% of that in *H. unifasciatus* and *H. perrieri* (Fig. 2).

## Discussion

### Resource shifts, range expansions and genetic diversity

The three *Helictopleurus* species that are presently common in cattle dung across Madagascar exhibit two strikingly different patterns of molecular diversity in CO1. *Helictopleurus neoamplicollis* and *H. marsyas* have very limited haplotype diversity and a single haplotype is present across Madagascar. The other few haplotypes differ by only one or two nucleotide changes from the common one, and the rare haplotypes mostly occur in the same region in north-western Madagascar. This pattern is most parsimoniously explained by the species having shifted to cattle dung within a small region in north-western Madagascar, where the haplotype diversity is presently highest, followed by rapid range expansion.

The pattern in *H. quadripunctatus* suggests a different process, a gradual change of diet across much of the original range, which may have covered most of southern Madagascar, where the species currently shows high haplotype diversity. This hypothesis is consistent with the ecology and current resource use of *H. quadripunctatus.* Unlike the other two species, which are hardly ever sampled with other types of resource than cattle dung, *H. quadripunctatus* is attracted in large numbers to human excrement and also to carrion both in open areas and in dry forests. Apparently, *H. quadripunctatus* has a broader diet than the other cattle dung-using species, and it is probable that cattle dung was gradually included in its diet in multiple populations across the original range. Nonetheless, the geographical distribution of haplotypes suggests that *H. quadripunctatus* has recently expanded its range relatively rapidly towards north, where just a single haplotype has been sampled in many localities.

One might think that range expansions following resource or host shift are commonplace, and they may be so, but putative examples are mostly anecdotal. For instance, Braschler & Hill (2007) suggested that current range expansion in the butterfly *Polygonia c-album* in northern Europe is facilitated not only by climate warming but also by altered host plant preference from *Humulus lupulus* to the more widespread and common *Ulmus glabra* and *Urtica dioica*. Somewhat stronger evidence has been reported for the tephritid fly *Tephritis conura*, which has been inferred to have colonized *Cirsium oleraceum* from populations using *Cirsium heterophyllum* (Diegisser *et al*. 2006). Genetic diversity was significantly lower in fly populations on *C. oleraceum*, and there was less spatial genetic structure in the populations sampled on this host species than on *C. heterophyllum*, suggesting range expansion following host shift to *C. oleraceum*. Definite cases of host shift-induced range expansion involve parasitic fungi that have shifted to domesticated plants (Zaffarano *et al.* 2008) and disease epidemics in general (Fisher *et al.* 2001), into which we may include HIV shifting to humans.

There are also well-documented cases with no range expansion following host shift even if the new host is more widely distributed. For instance, in the checkerspot butterfly *Euphydryas editha* in western USA, two endangered subspecies of *E. editha* have shifted to the introduced and widely distributed weed *Plantago lanceolata*, but in neither case has the host shift allowed the insects to break out of their highly restricted distributions (Singer *et al.* 2008). Apparently, there are other ecological factors that prevent range expansion, and the original range was not primarily restricted by the distribution of the host species. An intermediate example is provided by the well-studied tephritid fly *Eurosta solidaginis*, which has been inferred to have recently shifted to the new host plant *Solidago gigantea* from the ancestral host *Solidago altissima* (Waring *et al.* 1990; Craig *et al.* 1993). Populations using the latter host have greater genetic diversity and more spatial genetic structure than populations using the new host (Brown *et al.* 1996), but the fly populations on the new host have a limited range compared with the range of the host plant (W. Abrahamson, personal communication). Finally, there are well-documented examples of the reversed causality, host shift following range expansion that exposed the consumer to novel resources. Numerous such examples have been reported for introduced species in e.g. the context of biological control (Simberloff & Stiling 1996).

In conclusion, our results demonstrate that even a limited amount of sequence information, combined with spatially systematic sampling of multiple species, can provide strong evidence of host shift-related range dynamics.

### Ecological context of resource shifts in *Helictopleurus*

*Helictopleurus quadripunctatus*, *H. unifasciatus* and *H. perrieri* form a polytomous clade in molecular phylogeny (Wirta *et al.* 2008). The latter two species occur in dry forests in western Madagascar, while *H. quadripunctatus* may have originally inhabited dry forests in southern Madagascar, where it currently shows the highest genetic diversity. Concerning the original resource use of *H. quadripunctatus*, it may have used the excrements of the extinct lemurs, the largest of which weighed more than 100 kg (Burney *et al.* 2004). The lemurs went extinct in the past 2000 years following human arrival, but human excrements then provided a new resource not dissimilar to the excrements of the large-bodied lemurs. *Helictopleurus quadripunctatus* is a relatively large species (body length 10–11 mm), and it may hence not be able to breed on the excrements of the extant small species of lemurs. The close association between humans and cattle may have contributed to the resource shift to cattle dung.

**Figure 3:**
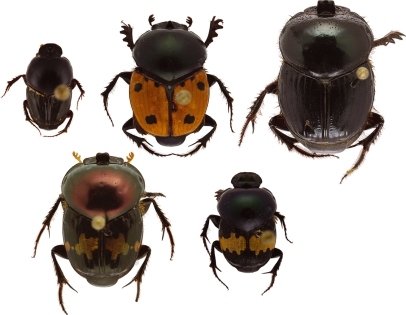
Upper row: *Helictopleurus neoamplicollis*, *Helictopleurus quadripunctatus* and *Helictopleurus marsyas*. Lower row: *Helictopleurus unifasciatus* and *Helictopleurus perrieri*.

One particular feature worth noting is the colouration of *H. quadripunctatus*, with bright yellow-red elytra with four black dots and shining dark green or dark blue pronotum depending on the region in Madagascar (Fig. 3). Such bright colouration is exceptional for the entire genus *Helictopleurus*, in which the species are either uniformly dark or with green, reddish or black pronotum and commonly spotted pattern on green, dark red or blackish elytra. *Helictopleurus quadripunctatus* is very conspicuous in the field, giving the impression of warning colours. It would be interesting to know how lemurs and insectivorous birds respond to *H. quadripunctatus*, and whether indeed the bright colouration would reduce predation risk in the open habitats.

*Helictopleurus marsyas* and *H. nicollei* are best considered as sister species or incipient sister species. There is a substantial difference in their CO1 sequences, considering the nearly complete lack of molecular diversity in CO1 in *H. marsyas* across Madagascar*.* The level of differentiation between *H. marsyas* and *H. nicollei* with 13 fixed nucleotide differences must be much older than 1500 years, the time of cattle introduction to Madagascar, and probably represents differentiation between populations on the east (*H. nicollei*) and the west coast (*H. marsyas*). At present, the difference in resource use and habitat selection will help keep the taxa separate. *Helictopleurus nicollei* presently occurs within a small area on the east coast, in small remaining fragments of wet forest. It may have previously had a greater range but must have suffered of the nearly complete deforestation of the lowland areas on the east coast, along with numerous other forest-living *Helictopleurus* species (Hanski *et al.* 2007). *Helictopleurus nicollei* is one of the few wet forest-dwelling *Helictopleurus* that have been sampled in small numbers in cattle dung pats (Rahagalala *et al.*, unpublished data). It is thus possible that there are some pre-adaptations in this species pair that have facilitated the resource shift in *H. marsyas.* One trait that may be of importance is simply the large size of the species, which has probably facilitated the resource shift to cattle dung (Wirta *et al.* 2008); both *H. nicollei* and *H. marsyas* belong to the largest extant *Helictopleurus* species (average body length 15 mm; Fig. 3).

The evident resource shift-induced expansion in the geographical ranges of *H. neoamplicollis* and *H. marsyas*, and to a lesser extent of *H. quadripuntatus*, has implications for the factors affecting range size in the forest-dwelling species. The relatively small ranges of the latter are not caused by the geographical distribution of their resources, as most species have a broad diet including many types of dung and even carrion (Wirta *et al.* 2008). Equally, the fact that the three cattle dung-using *Helictopleurus* have spread across entire Madagascar implies that the ranges of the remaining species are unlikely to be restricted by climatic factors, with the caveat that the wet forest species are restricted to this biome. Instead, the ranges of most *Helictopleurus* are likely to be limited by strong interspecific interactions. The forest species constitute highly competitive communities (H. Viljanen, H. Wirta, O. Montreuil, P. Rahagalala, S. Johnson & I. Hanski, unpublished data) as dung beetles do elsewhere in the tropics (Hanski & Cambefort 1991). The Malagasy dung beetle communities exhibit very high β diversity. For instance, two well-studied communities in the Ranomafana and Masoala National Parks on the east coast, separated by 650 km, have 36 and 31 species respectively, but they have only four species in common (Viljanen *et al.*, unpublished data), although there is no real difference in the forest habitat. Apparently, range expansion is hindered by the presence of ecologically similar competitors in the neighbouring areas. The shift to cattle dung by *H. neoamplicollis*, *H. marsyas* and *H. quadripunctatus* relaxed resource competition and thereby allowed these species to expand their ranges and to become exceedingly common. There is one possibly uncommon cattle dung-using species, *H. sinuatocornis*, which we have not analysed in this paper because of lack of material, but this species occurs only at high elevations and is poorly sampled (Wirta *et al.* 2008).

The very limited haplotype diversities in *H. neoamplicollis* and *H. marsyas* in comparison with the forest-living species suggests that the lineages of the former species that now occur in cattle dung across Madagascar had very limited ranges at the time of resource shift, possibly due to extensive deforestation. Hanski *et al.* (2007) found that the extent of forest loss in the past 50 years (for which data are available) within the historical ranges of particular *Helictopleurus* species was the best predictor of whether the species was caught or not in Madagascar-wide sampling in 2002–2006. There are *c*. 20 *Helictopleurus* species that have not been sampled in the past decade and mostly not for the past 50 years; these species may already be extinct or effectively extinct due to deforestation. The shift to cattle dung may have saved *H. marsyas* and *H. neoamplicollis* from imminent extinction.
